# Expanding the Genetic Landscape of Congenital Stationary Night Blindness Through the Analysis of Consanguineous Pakistani Families

**DOI:** 10.3390/genes17050539

**Published:** 2026-05-01

**Authors:** Razia Parveen, Muhammad Iqbal, Shahbaz Khan, Abdur Rashid, Helen Nabiryo Frederiksen, Sergey Oreshkov, Ghulam Mustafa, Muhammad Asif Naeem, Hafiz Muhammad Azhar Baig, Muhammad Ansar

**Affiliations:** 1 Department of Biochemistry and Molecular Biology, Institute of Biochemistry, Biotechnology and Bioinformatics, The Islamia University of Bahawalpur, Bahawalpur 63100, Pakistan; raziamschemistry@gmail.com (R.P.);; 2 Department of Biotechnology, Institute of Biochemistry, Biotechnology and Bioinformatics, The Islamia University of Bahawalpur, Bahawalpur 63100, Pakistan; iqbal.khawaja@iub.edu.pk (M.I.); gmqau@hotmail.com (G.M.); 3 Department of Ophthalmology, University of Lausanne, Jules Gonin Eye Hospital, Fondation Asile des Aveugles, 1004 Lausanne, Switzerland; rashid.abdur@fa2.ch (A.R.); helen.frederiksen@fa2.ch (H.N.F.); sergey.oreshkov@fa2.ch (S.O.); 4 Centre for Applied Molecular Biology, University of the Punjab, Lahore 53700, Pakistan; asif.camb@pu.edu.pk; 5 Jacobs Retina Center, Shiley Eye Institute, University of California San Diego, La Jolla, CA 92039, USA; 6 Viterbi Family Department of Ophthalmology, Shiley Eye Institute, University of California San Diego, La Jolla, CA 92039, USA; 7 Advanced Molecular Genetics and Genomics Disease Research and Treatment Centre, Dow University of Health Sciences, Sindh 74200, Pakistan

**Keywords:** consanguinity, CSNB, *TRPM1*, *GPR179*, *SAG*, *SLC24A1*, *GRK1*, exome sequencing

## Abstract

**Background/Objectives**: The current study was designed to identify the underlying genetic causes of congenital stationary night blindness (CSNB) in the indigenous consanguineous families from the Southern Punjab region of Pakistan, a population where the inherited retinal disorders are relatively common. **Methods**: A detailed questionnaire and medical examination were done to check the presence of CSNB in the affected individuals of the enrolled families. Whole-exome sequencing (WES) was performed to identify the pathogenic variants, followed by segregation analyses to confirm the segregation of the identified variants with the disease phenotype in the available affected individuals of the families. **Results**: We identified two novel and three known pathogenic variants in *SAG*, *GRK1*, *TRPM1*, *SLC24A1*, and *GPR179,* having established roles in CSNB. Two novel variants, NM_001252020.1 (p.Gly1020Arg) and NM_001004334.3 (p.Trp508Ter), were identified, and their segregation was confirmed in two families, PKIURP102 and PKIURP564, respectively. NM_002929.3 (p.Arg19Ter) and NM_001301032.1 (p.Phe538CysfsTer23) were the reported variants identified in PKIURP17 and PKIURP528 families, respectively. NM_000541.5 (p.Glu306Ter) was identified in two independent families, PKIURP552 and PKIURP565. **Conclusions**: Identification of five pathogenic variants in five different genes shows the genetic heterogeneity of CSNB in Pakistani patients. Our findings also expand the mutational spectrum of CSNB in the Pakistani population and may help in the identification of mutational hotspots and may help in the genetic diagnosis of CSNB in consanguineous populations.

## 1. Introduction

Congenital stationary night blindness (CSNB), first described by Florent Cunier in 1838, is a genetically heterogeneous group of disorders that primarily affect photoreceptor cells, bipolar cells, or the retinal pigment epithelium (RPE) [1,2,3]. It is a non-progressive inherited retinal disorder caused by pathogenic or likely pathogenic variants in genes that are essential for normal retinal signal transduction. Rather than resulting from photoreceptor degeneration, CSNB arises from defects in proteins that regulate phototransduction, synaptic vesicle release, or post-synaptic signaling between photoreceptors and ON-bipolar cells. Variants affecting phototransduction components disrupt the timely deactivation of rod responses, while defects in synaptic or post-synaptic proteins impair signal transmission from rods to bipolar cells, leading to selective dysfunction of the rod pathway under low-light conditions. These gene-specific molecular defects directly explain the characteristic functional abnormalities observed in CSNB despite preserved retinal structure. Genetic associations are established by identifying rare deleterious variants in biologically relevant genes and confirming their segregation with the disease phenotype within affected families, thereby linking molecular dysfunction to clinical presentation [3,4,5]. CSNB is characterized by impaired night vision or delayed dark adaptation [6]. While most patients report difficulty seeing in low-light conditions, a subset may also experience photophobia [7]. Additional clinical features may include myopia, strabismus, nystagmus, and reduced visual acuity [2]. However, because night vision is a subjective experience, especially for individuals living in well-lit urban environments, symptoms may be overlooked or underreported.

The global prevalence of CSNB varies significantly across populations and is likely underreported due to diagnostic limitations. For instance, in Northern Europe, the prevalence is estimated at 0.34 per 100,000, but this figure may not reflect the true burden of disease due to underdiagnosis and lack of routine screening [8]. The rarity and clinical heterogeneity of CSNB further complicate accurate epidemiological assessments, particularly in regions with limited access to electroretinography (ERG) and genetic testing [9].

CSNB is broadly classified into two categories based on fundus appearance: forms with fundus abnormalities and those without any fundus abnormality [3]. The former includes Oguchi disease and fundus albipunctatus (FAP), while the latter encompasses complete and incomplete Schubert–Bornschein types and Riggs type, distinguished by characteristic ERG findings [10,11]. Oguchi disease is a non-progressive retinal disorder with genetic heterogeneity, caused by autosomal recessive mutations in *GRK1* or *SAG* [12,13]. It presents with a distinctive golden-yellow discoloration of the fundus, particularly in the posterior pole and peripheral retina under light-adapted conditions. This discoloration disappears after prolonged dark adaptation, a phenomenon known as the Mizuo–Nakamura sign, which is considered a hallmark of the disease [11,14,15]. FAP, on the other hand, is characterized by small white retinal spots believed to contain precursors of 11-cis-retinal and is primarily associated with recessive mutations in *RDH5* [11,16].

To date, at least 19 genes have been implicated in CSNB, including *CABP4*, *CACNA1F*, *CACNA2D4*, *GNAT1*, *GPR179*, *GRK1*, *GRM6*, *LRIT3*, *NYX*, *PDE6B*, *RDH5*, *RHO*, *RLBP1*, *RPE65*, *SAG*, *SLC24A1*, *TRPM1*, *GNB3*, and *RIMS2* [3,17,18]. Mutations in *GUCY2D* have been associated with congenital stationary night blindness that may progress to mild retinitis pigmentosa (RP) [19]. A dual phenotype of CSNB and cone-rod dystrophy has also been reported in siblings carrying mutations in both *GNAT1* and *ABCA4* [20].

Consanguineous populations provide a unique opportunity to investigate autosomal recessive Mendelian disorders, as they increase the likelihood of homozygosity for rare variants. Pakistan is among the most consanguineous countries globally, with over 50% of marriages occurring between cousins and rates reaching up to 80% in certain regions [21]. The southern Punjab region is one such area, characterized by high consanguinity and limited awareness of its genetic risks. While numerous genes and mutations associated with inherited disorders have been reported in Pakistani families, most originate from central Punjab, Khyber Pakhtunkhwa and Sindh provinces [22,23,24]. A previous study from southern Punjab has already documented CSNB-related variants [6], and the present work represents the second report from this region.

In this study, we analyzed six autosomal recessive consanguineous families confirmed clinically with CSNB phenotypes from the southern Punjab region of Pakistan and identified novel and known variants in CSNB genes.

## 2. Materials and Method

### 2.1. Ethical Approval

This study was approved by the Bioethics Committee of the Islamia University of Bahawalpur, Pakistan.

### 2.2. Enrollment of Families

All families included in this study were recruited from the southern region of Punjab Province, Pakistan. Enrollment criteria included at least two affected individuals per family, confirmed parental consanguinity, and inheritance patterns consistent with autosomal recessive based on pedigree analysis. These families are part of a broader cohort of families with inherited retinal dystrophies; however, the six pedigrees analyzed here were the only ones exhibiting definitive CSNB phenotypes. To exclude syndromic forms of retinal disease, a detailed questionnaire was designed which had questions regarding the presence of any other abnormalities, including but not limited to the presence of intellectual disability, obesity, cataract and glaucoma. Written informed consent was obtained from all participants or their parents/legal guardians for their voluntary participation in this study. The family PKIURP17 and PKIURP528 had six affected individuals in each family, while families PKIURP102, PKIURP552, PKIURP564 and PKIURP565 had three affected individuals in each family. All the affected individuals in family PKIURP552 were females in a single loop. In family PKIURP564, affected individuals were in two loops, where individual II.1 was not alive at the time of enrollment. The family PKIURP565 had affected individuals in two loops, where only one individual, IV.1, was alive at the time of sampling.

### 2.3. Clinical Examination

Clinical examination of affected individuals from three families, PKIURP552, PKIURP564 and PKIURP565, was made by the ophthalmologist, consisting of medical history, family history and fundoscopy to confirm clinical diagnosis. The remaining three families, PKIURP17, PKIURP102 and PKIURP528, underwent diagnosis based on available medical history and descriptive phenotype as the families were residents of remote areas that were difficult for an ophthalmologist to approach.

### 2.4. Sample Collection and DNA Extraction

From all the enrollees of the families, 10 mL of venous blood was obtained in 50 milliliter Falcon^®^ conical centrifuge tubes (Corning Life Sciences, Tewksbury, MA, USA) already having 400 microliters of 0.5 M Ethylenediaminetetraacetic acid (EDTA) as an anticoagulant (Merck KG Sigma-Aldrich, Darmstadt, Germany and its aquous solution was autoclaved before use). Tubes were properly labeled and were stored at −20 °C. Standard techniques were used to obtain genomic DNA from white blood cells [25]. DNA was assessed both quantitatively and qualitatively using 0.8% agarose gel electrophoresis, NanoQ Plus Spectrophotometer (K Lab, Daejeon, Republic of Korea), and UV Transilluminator (Fisher Scientific, Hampton, NH, USA).

### 2.5. Whole-Exome Sequencing

Probands from all six families underwent whole-exome sequencing (WES) utilizing Twist Comprehensive Exome Panel (Twist Bioscience, South San Francisco, CA, USA). A HiSeq 4000 instrument (Illumina, San Diego, CA, USA) was used for sequencing with an average coverage of 100–120× for each nucleotide position. Novoalign v3.08.00 (Novacraft Technologies, Selangor, Malaysia) was used to map raw reads to the human genome reference sequence (build hg19). HaplotypeCaller v4.0.3.0 (GATK) was used to recalibrate the Base Quality score. Removal of duplicates was done using Picard v2.14.0 (SNAPSHOT). Small indels and SNVs were identified by using GATK v4.0 [26].

### 2.6. Bioinformatic Analyses

Minor allele frequencies for all identified variants were checked using the gnomAD database [27]. Conservation of variants was assessed by using GERP++ [28], PhastCons100way score [29] and PhyloP100way score [30]. Pathogenicity of all variants was predicted using ANNOVAR (v2020-06-07), CADD phred (v1.7), DANN scoring (obtained from dbNSFP v5.3.1) and an in-house pipeline [31]. Variants that passed the filtering stages were assessed using the ACMG standards and variant interpretation guidelines. The HGMD, ClinVar, and LOVD databases were searched to find previously known harmful variants. Several in silico techniques were used to assess novel variations for their possible effects on protein function. Protein–protein interaction was assessed using the STRING database [32].

### 2.7. Sanger’s Validation and Segregation Test

Sanger’s sequencing was used to confirm segregation of each potential pathogenic variation in the families. The Primer3 plus program [33] was used to design primers that would amplify the area spanning the variation with a minimum of 50 flanking base pairs. Depending on the availability of DNA samples, proband, other affected individuals, and non-affected members of each family underwent Sanger’s sequencing and segregation analysis.

## 3. Results

### 3.1. Clinical Findings

**Figure 1:**
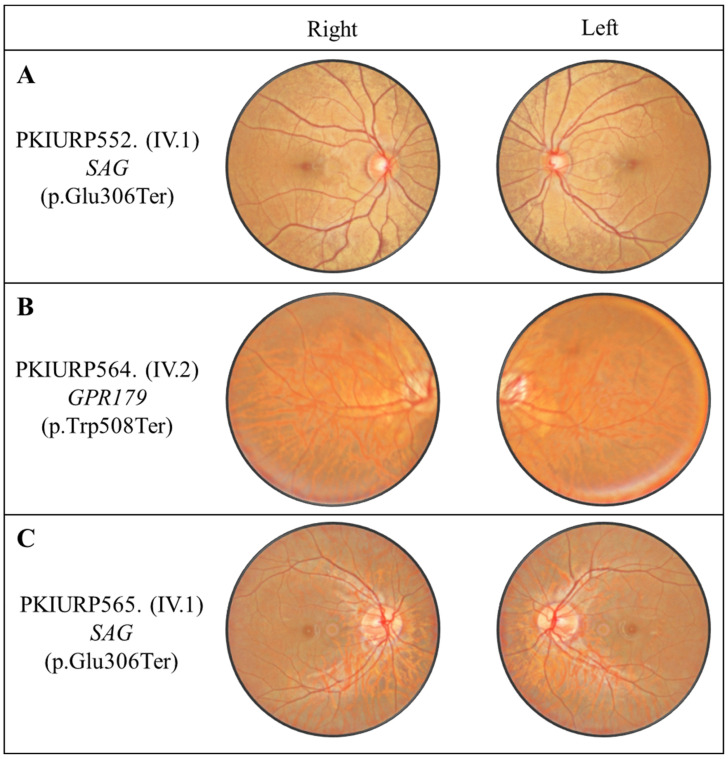
Fundus photographs of both eyes of probands from families. (**A**) PKIURP552. (**B**) PKIURP564. (**C**) PKIURP565.

Medical history and clinical pictures of affected individuals of family PKIURP17, PKIURP102 and PKIURP528 showed that all the affected individuals had congenital stationary night blindness. On the other hand, the Ophthalmoscopic examination, in addition to a descriptive picture of affected individuals from family PKIURP552, PKIURP564 and PKIURP565, is suggestive of CSNB (Figure 1).

The initial symptom in affected individuals of all families was night blindness that began at an early age. Visual field and color perception were normal and night blindness was non-progressive in all affected individuals (Table 1). The Mizuo–Nakamura phenomenon, a golden coloring of the fundus that vanishes in a fully dark-adapted state and returns soon after the onset of light, is prominent in affected individuals. Fundus images of proband IV:1 from family PKIURP552 showed a distinctive appearance of the Mizuo–Nakamura phenomenon of Oguchi disease (Figure 1A). In family PKIURP564, fundus images of the Proband (IV:2) showed sectoral changes in retinal pigment epithelium (RPE), attenuated arteries, and a slightly tilted waxy optic disc (Figure 1B). The individual IV.1 from PKIURP565 showed fundus affected with CSNB (Figure 1C).

### 3.2. Molecular Findings

**Figure 2:**
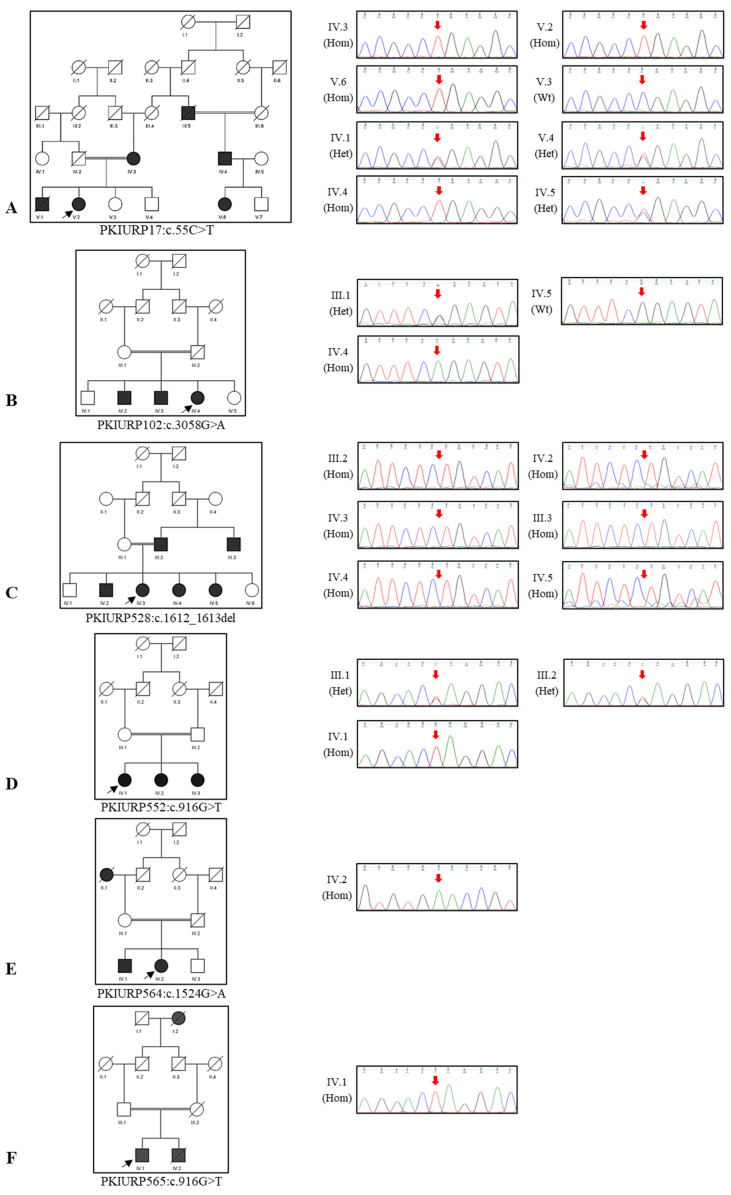
Segregation analysis of the variants identified within the pedigrees analyzed. In the left, pedigrees are given showing circles as females while squares representing males. A single horizontal line in between male and female indicates a marriage while a double such line indicates consanguineous marriage. A crossed square or circle means the individual is deceased. Eacch individual in the pedigree is denoted with a unique identity in which Roman number represents the generation of the family while integer indicates number of that individual in the family. In the right, electropherograms are shown representing results of Sanger sequencing of target DNA region. Probands from each family are indicated by the arrow sign in the pedigrees. Mutation identified through WES of proband is mentioned below each pedigree. In the right, findings of Sanger sequencing are shown in the form of electropherograms with individual identity as per the pedigree and the state of zygosity; Hom:Homozygous, Het:Hetrozygous, Wt:Wildtype. The DNA sequence of target region in the electropherograms is represented with peaks of various colours, each peak representing a specific nucleotide; Green:A, Blue: C, Red:T, Black:G, while the red arrow points to the site of mutation. (**A**): PKIURP17. (**B**): PKIURP102. (**C**): PKIURP528. (**D**): PKIURP552. (**E**): PKIURP564. (**F**): PKIURP565.

WES analysis of probands from all six families revealed five DNA variants in five IRD-related genes. In family PKIURP17, a nonsense homozygous mutation c.55C>T (p.Arg19Ter) was observed in *GRK1*. The Sanger sequencing confirmed the presence of this biallelic variant in all affected individuals IV.3, IV.4, V.2 and V.6, while phenotypically normal individuals IV.1, IV.5 and V.4 had a heterozygous mutation and V.3 was found to be homozygous normal (Figure 2A). In family PKIURP102, a homozygous missense mutation c.3058G>A (p.Gly1020Arg) was found in *TRPM1.* The Sanger sequencing confirmed the said variant in IV.4, while her mother III.1 was found to be heterozygous and a sibling IV.5 was homozygous normal (Figure 2B).

In family PKIURP528, a homozygous frameshift deletion c.1612_1613del (p.Phe538CysfsTer23) was found in *SLC24A1*. The Sanger sequencing confirmed the presence of the homozygous deletion in all six affected individuals III.2, III.3, IV.2, IV.3, IV.4 and IV.5 (Figure 2C). In family PKIURP564, a homozygous nonsense variant c.1524G>A (p.Trp508Ter) was found in *GPR179,* which was confirmed by Sanger sequencing in individual IV.2 (Figure 2E). In families PKIURP552 and PKIURP565, a common homozygous stop-gain variant c.916G>T (p.Glu306Ter) was found in *SAG*. In family PKIURP552, the variant (c.916G>T) was confirmed homozygous by Sanger sequencing in affected individual IV.1, while her parents, III.1 and III.2, were found to be heterozygous (Figure 2D). In family PKIURP565, affected individual IV.1 was confirmed to be homozygous for variant c.916G>T (Figure 2F).

### 3.3. In Silico Prediction

**Figure 3:**
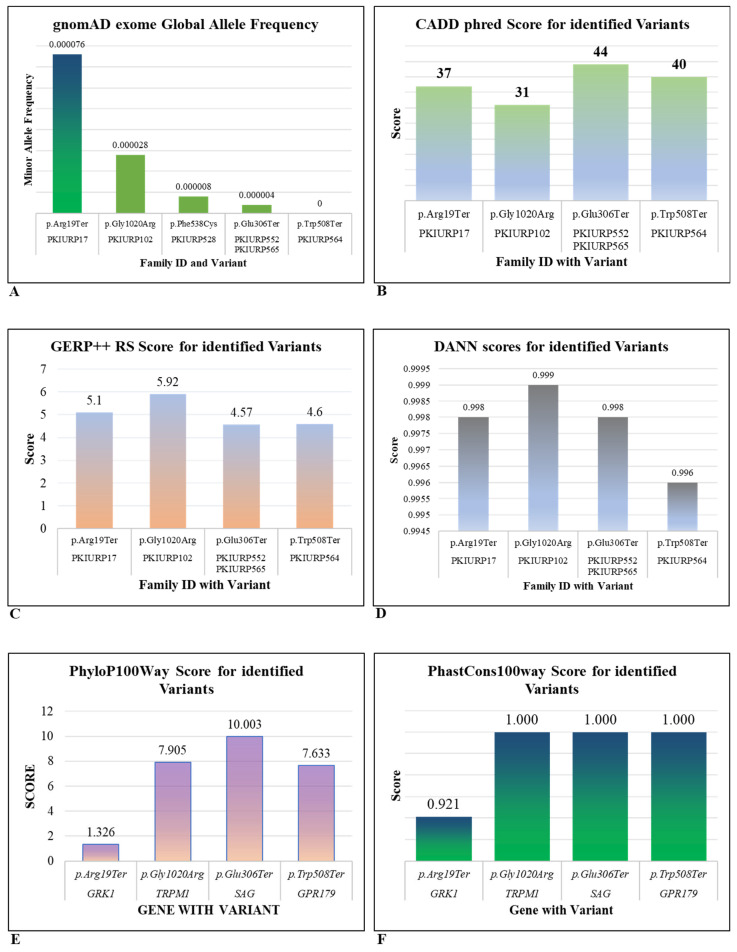
In silico prediction of pathogenicity and conservation for identified variants. (**A**) gnomAD Exome Allele Frequency. (**B**) CADD phred Scores. (**C**) GERP++ RS Scores. (**D**) DANN Scores. (**E**) PhyloP100way Scores. (**F**) PhastCons100way Scores.

In addition to Sanger sequencing, in silico prediction tools also suggested the deleterious nature of identified variants. The minor allele frequency obtained from the gnomAD database for all populations and ancestors throughout the world, by taking 1 in 1000 populations as a threshold, suggests the exceptionality of all identified variants and their pathogenic behaviors (Figure 3A). The CADD phred score calculated for identified variants, except the deletion mutation, is found above the threshold value of 30, meaning all variants are among the top 0.1% of all possible deleterious substitutions in the human genome and are highly likely to be damaging (Figure 3B).

The Genomic Evolutionary Rate Profiling Rejected Substitution (GERP++ RS) score, which provides evolutionary constraint against a specific position in DNA, was estimated for identified DNA variants. This score ranges from −12.3 to 6.17, where a higher score refers to a more conserved site. All estimated scores for identified variants were near the higher value, suggesting their high conservation and damaging effect on any change in that very nucleotide (Figure 3C). DANN scoring was estimated for identified substitution mutations that are based on a Deep Neural Network, where the score ranges from 0 to 1. A higher score indicates a higher likelihood of the deleterious nature of a variant. DANN scores for all identified variants were near 1, indicating their damaging nature (Figure 3D). PhastCons100way scores and PhyloP100way scores estimated for all identified variants indicated their high conservation as suggested by their estimated scores near the upper limits (Figure 3E,F). Moreover, except for the deletion mutation in *SLC24A1*, the rest of the four variants were found deleterious in other in silico prediction tools, like LRT scoring, BayesDel noAF prediction and fathmm-MKL coding (Table 2).

### 3.4. String Analysis

**Figure 4:**
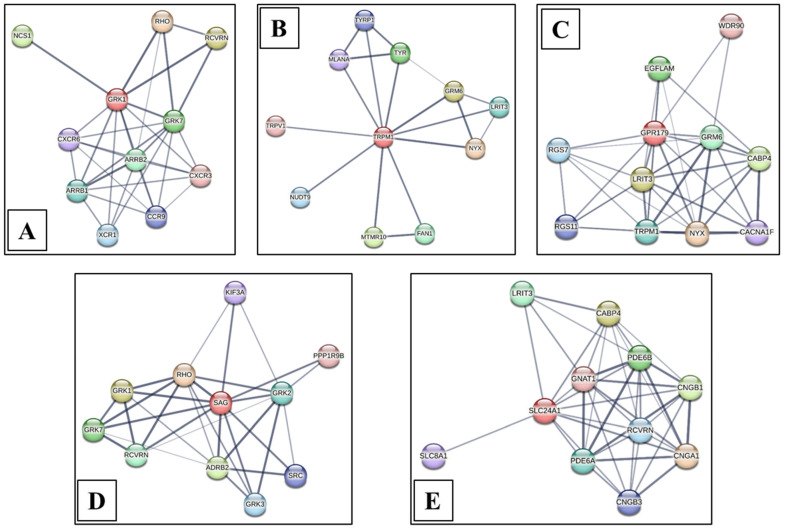
Interaction analyses of identified proteins with other proteins of visual function. Nodes indicate protein names, while edges joining the nodes indicate their in-between associations, keeping the identified protein as central. The strength of edges indicates the strength of association. (**A**) GRK1. (**B**) TRPM1. (**C**) GPR179. (**D**) SAG. (**E**) SLC24A1.

As a critical regulator in rod photoreceptors to mediate phosphorylation, a defect in GRK1 would disrupt its normal function, preventing proper rhodopsin inactivation. Consequently, downstream associations with RCVRN, which modulates GRK1 activity in a calcium-dependent manner, and with GRK7 (the cone counterpart), would be functionally uncoupled, impairing both rod and cone phototransduction recovery. Impaired recruitment of ARRB1 and ARRB2 would further compromise receptor desensitization and internalization, leading to sustained phototransduction, altered trafficking of associated chemokine receptors like CXCR3, CXCR6 and CCR9, ultimately disrupted visual signaling, aberrant inflammatory signaling in non-ocular tissues where these pathways overlap and heightened susceptibility to light-induced retinal degeneration (Figure 4A).

As a key downstream effector in the retinal ON-bipolar cell pathway, a defect in TRPM1 would disrupt this ion channel’s ability to conduct depolarizing currents, effectively uncoupling the signal transmission from GRM6 activation. Consequently, the functional associations with LRIT3 and NYX become pathological, as neither can compensate for absent or nonfunctional TRPM1 channels, leading to a complete failure of ON-bipolar cell responses to light. This disruption secondarily affects downstream visual signaling, with no direct functional impact on melanosomal proteins like TYRP1, TYR and MLANA despite their co-visualization in the network, thereby manifesting clinically as impaired night vision, loss of contrast sensitivity and the characteristic finding of photoreceptor with abolished ON-bipolar cell function (Figure 4B).

GPR179 is essential for the proper clustering and anchoring of GRM6 and its downstream signaling cascade at the dendritic tips of ON-bipolar cells, where it facilitates the physical coupling of light-induced glutamate signaling to TRPM1 channel activation. A defect in GPR179 would disrupt the assembly and stability of this macromolecular complex, leading to mislocalization or impaired function of GRM6, LRIT3, and the associated RGS7/RGS11 heterotrimer, which normally fine-tunes the kinetics of G-protein signaling. Consequently, the functional linkage to TRPM1 is severed, preventing the depolarizing cation influx required for ON-bipolar cell responses to light. This breakdown in signaling propagation also affects the functional integrity of associated proteins like NYX, which interacts within the same complex, and indirectly alters calcium-mediated modulation via CACNA1F and CABP4, culminating in abolished ON-pathway visual transmission, diminished night vision, and the characteristic electroretinographic changes despite preserved photoreceptor function (Figure 4C).

SAG is responsible for binding to light-activated, GRK1/GRK7-phosphorylated rhodopsin, thereby quenching its activity and preventing further transduction signaling. A defect in SAG would disrupt high-affinity arrestin binding to phosphorylated rhodopsin, leading to prolonged rhodopsin activity and sustained phototransduction even in bright light. This functional impairment directly compromises its interaction with GRK1 and GRK7, as the kinase-mediated phosphorylation becomes ineffective in achieving signal termination without competent arrestin; the association with RHO becomes pathological because the receptor cannot be properly inactivated. Consequently, the downstream linkage to ADRB2 and other interacting partners like KIF3A and SRC is disturbed, impairing receptor recycling, desensitization of non-visual GPCRs and overall retinal adaptation, manifesting clinically as congenital stationary night blindness with normal photoreceptor structure but markedly delayed rod recovery kinetics on electroretinography (Figure 4D).

SLC24A1 is a critical regulator of calcium homeostasis and recovery in photoreceptors. As the primary calcium extruder in rod photoreceptor outer segments, SLC24A1 mediates the rapid decline of intracellular calcium following light exposure, a process essential for terminating phototransduction and enabling dark adaptation via calcium-sensitive proteins like recoverin and guanylate cyclase-activating proteins. A defect in SLC24A1 would impair calcium extrusion, leading to persistently elevated intracellular calcium during light responses. This disruption directly compromises the functional association with RCVRN, which normally adjusts guanylate cyclase activity in a calcium-dependent manner, thereby preventing timely recovery of cyclic GMP levels and reopening of CNGA1/CNGB1 channels. Consequently, the coordinated interplay with GNAT1, PDE6A/PDE6B, and the CNG channels becomes uncoupled, resulting in delayed photoresponse termination, impaired dark adaptation and altered calcium-mediated modulation of downstream targets such as SLC8A1, culminating in defective visual signal transmission, photoreceptor stress and retinal degeneration (Figure 4E).

## 4. Discussion

CSNB is a clinically and genetically heterogeneous retinal disorder characterized by non-progressive impairment of scotopic vision, often accompanied by nystagmus and myopia [3]. In the present study, we analyzed six consanguineous families and identified five pathogenic variants in genes implicated in CSNB: *GRK1*, *TRPM1*, *SLC24A1*, *SAG*, and *GPR179*. Each of these genes plays a critical role in retinal signal transduction, and their disruption leads to distinct molecular consequences.

In family PKIURP17, we identified a homozygous nonsense variant (p.Arg19Ter) in *GRK1*. -This gene, positioned on chromosome 13q34, encodes a serine/threonine protein kinase of 563 amino acids [34,35]. The protein contains a central AGC kinase catalytic domain embedded within a regulator of G-protein signaling domain, which facilitates ATP binding and substrate phosphorylation [36]. Pathogenic variants in *GRK1* are well known to cause Oguchi disease type II, and this condition is more frequently reported in patients of European descent [9]. Although a missense mutation in *GRK1* has previously been reported in a Pakistani family [37], the variant identified in our study (p.Arg19Ter) occurs near the start of the protein, resulting in early truncation and complete loss of GRK1 function. Importantly, p.Arg19Ter has also been documented as pathogenic in another consanguineous Pakistani family [38], further supporting the role of this variant in disease-causing.

Our finding is consistent with the previously reported truncating variants, including p.Glu48ProfsTer82 and p.Leu324ArgfsTer62, which support the idea that loss of function may be a key disease causing mechanism in *GRK1*-associated Oguchi disease [39,40]. In contrast, the reported missense variant p.Arg438Cys highlights the allelic heterogeneity observed in *GRK1*-associated Oguchi disease.

In family PKIURP102, affected individuals carried a homozygous missense variant, p.Gly1020Arg, in *TRPM1*, which encodes a non-selective cation channel essential for light-induced depolarization in ON-bipolar retinal cells [41]. *TRPM1* is a complex gene spanning 58 kb and 27 exons, producing multiple isoforms through alternative splicing [42,43,44]. *TRPM1* mutations are recognized as the leading genetic cause of CSNB in the Indian population [45]. The mutational spectrum of *TRPM1* includes diverse pathogenic variants, such as missense, splice-site, and stop-gain mutations [46]. The identification of p.Gly1020Arg in a family of Pakistani origin expands the spectrum of *TRPM1*-associated CSNB reported in South Asian populations. *TRPM1* variants have been more commonly implicated in CSNB in Israeli and Palestinian populations, including deletions involving exons 3–7 described in an Ashkenazi Jewish family, and a nonsense variant, p.Arg877Ter identified in a Palestinian family, however reports from Pakistani cohorts remain limited [47,48]. Our findings, therefore, contribute additional evidence of population-specific variation and support the inclusion of *TRPM1* in genetic screening strategies for CSNB in South Asian patients.

In family PKIURP528, we identified a two-base-pair homozygous deletion, c.1612_1613del (p.Phe538CysfsTer23) in *SLC24A1*, which encodes a potassium-dependent sodium/calcium exchanger critical for calcium extrusion in rod photoreceptors during light adaptation. Loss-of-function mutations in this gene disrupt calcium homeostasis, impairing visual signaling [49,50]. Globally, pathogenic variants in *SLC24A1* are predominantly frameshift and stop-gain mutations, all leading to truncated proteins and supporting a common mechanism of disease [51]. Interestingly, a similar two-base-pair deletion has been reported in another family from the same region of Pakistan, suggesting a possible regional founder effect [49]. Such clustering of rare variants may reflect shared ancestry or limited genetic diversity in isolated populations. Although this frameshift mutation is classified as pathogenic, its recurrence in Pakistani families remains underreported, emphasizing the need for population-specific genetic studies. Documenting these cases broadens the mutational spectrum of *SLC24A1* and provides insights into regional genetic architecture. A similar homozygous deletion, identified in our study, c.1691_1693del (p.Phe564del), was previously reported by Yusuf et al. in 2023 [52], indicating that biallelic disruption of this region can independently cause disease. By contrast, Neuillé et al. in 2016 [51], later supported by Sundaramurthy et al. in 2025 [45], described affected individuals with compound heterozygous variants, c.1691_1693del and c.3291_3294del, resulting in p.(Phe564del) and p.(Val1099GlufsTer31), respectively. Together, these observations suggest that disease results from markedly reduced or absent functional protein, whether due to a homozygous truncating variant or the combined impact of two deleterious alleles.

In the current study, we also identified a homozygous variant (p.Glu306Ter) in two independent families, PKIURP552 and PKIURP565, in *SAG*. All available affected individuals exhibit an Oguchi phenotype. SAG plays a key regulatory role in the phototransduction cascade and is predominantly expressed in rod photoreceptors [53]. Mutations in *SAG* are most commonly associated with recessive forms of CSNB, although dominant and compound heterozygous variants have also been reported, indicating a broader inheritance spectrum of *SAG*-associated Oguchi disease [54,55,56]. Previous studies have also documented *SAG* variants in Pakistani families [6,57]. This variant has been reported in a patient of a consanguineous family having Pakistani origin, confirming its pathogenicity in this population [57]. The recurrence of this variant in families shows that this variant may be a recurrent variant in families of Oguchi disease from the southern Punjab region of Pakistan and may serve as a hotspot mutation in the families of Oguchi disease type 1.

In family PKIURP564, affected individuals carried a biallelic stop-gain variant (p.Trp508Ter) in *GPR179*. This gene encodes an orphan G-protein-coupled receptor that plays a crucial role in ON-bipolar cell signaling [58,59]. The protein consists of 2367 amino acids organized into distinct regions, including an N-terminal extracellular domain, a seven-transmembrane (7TM) segment and an intracellular C-terminal domain [60]. The p.Trp508Ter variant introduces a premature stop codon within the extracellular region, leading to truncation before the 7TM domain. As a result, the protein lacks both the 7TM domain and the C-terminal intracellular region, which are critical for membrane localization and signal transduction. This structural disruption likely causes complete loss of function, consistent with the pathogenic mechanism underlying *GPR179*-associated CSNB. Variants in *GPR179* have been well documented in CSNB across multiple studies [45,60,61]. Pathogenic variants in *GPR179,* including the nonsense variant, p.Arg525Ter, and the missense variant p.Pro604Leu have already been reported [60,62]. While these studies highlight the role of both truncating and missense variants in *GPR179*-associated disease, the identification of p.Trp508Ter extends the known genetic diversity of this gene and represents, to the best of our knowledge, the first report of this variant from a Pakistani family. This finding emphasizes the importance of evaluating *GPR179* in genetically unexplored populations and contributes to a broader understanding of the molecular basis of congenital stationary night blindness.

A key limitation of this study lies in the clinical characterization of the affected individuals. Comprehensive assessments, such as ERG and advanced imaging techniques, such as OCT and fundus autofluorescence, were not performed because of the unavailability of these facilities in the region. Additionally, the Mizuo–Nakamura phenomenon could not be documented in the probands of PKIURP17, PKIURP102, PKIURP528, and PKIURP564. The clinical diagnosis relied solely on medical history and questionnaire responses. The stable nature of night blindness in these cases supported a diagnosis of CSNB rather than RP. In the probands of families PKIURP552 and PKIURP565, fundus photographs were consistent with phenotypes of Oguchi disease, showing no hallmark RP features such as bone spicule pigmentation, vascular narrowing, or optic disc pallor. Importantly, the molecular findings aligned with the available clinical data for all examined probands. In summary, molecular analysis of six Pakistani families with CSNB revealed two novel pathogenic variants in *TRPM1* and *GPR179*, and three reported variants in *GRK1*, *SAG*, and *SLC24A1,* thereby expanding the known mutational spectrum of this rare retinal disorder.

## Figures and Tables

**Table 1:** Description of enrolled affected individuals of families.

Family ID	Individual ID	Gender	Age	Onset and Initial Signs/Symptoms
**PKIURP17**	IV.3	Female	45 Year	Early onset, non-progressive night blindness
	IV.4	Male	49 Year	Early onset, non-progressive night blindness
	V.2 *	Female	18 Year	Early onset, non-progressive night blindness
	V.6	Female	16 Year	Early onset, non-progressive night blindness
**PKIURP102**	IV.4 *	Female	19 Year	Early onset, non-progressive night blindness
**PKIURP528**	III.2	Male	38 Year	Early onset, non-progressive night blindness
	III.3	Male	41 Year	Early onset, non-progressive night blindness
	IV.2	Male	21 Year	Early onset, non-progressive night blindness
	IV.3 *	Female	19 Year	Early onset, non-progressive night blindness
	IV.4	Female	17 Year	Early onset, non-progressive night blindness
	IV.5	Female	15 Year	Early onset, non-progressive night blindness
**PKIURP552**	IV.1 *	Female	14 Year	Early onset, non-progressive night blindness
**PKIURP564**	IV.2 *	Female	33 Year	Early onset, non-progressive night blindness
**PKIURP565**	IV.1 *	Male	13 Year	Early onset, non-progressive night blindness

Asterisk (*) with the individual ID represents a proband.

**Table 2:** Allele frequency and in silico prediction of identified DNA variants.

Family ID	Gene	Variant	LRT Prediction	BayesDel noAF Prediction	Fathmm-MKL Coding Prediction
PKIURP17	*GRK1*	c.55C>T p.Arg19Ter	D	D	D
PKIURP102	*TRPM1*	c.3007G>A p.Gly1020Arg	D	D	D
PKIURP552	*SAG*	c.916G>T p.Glu306Ter	D	D	D
PKIURP565					
PKIURP564	*GPR179*	c.1524G>A p.Trp508Ter	D	D	D

The letter ‘D’ refers to ‘deleterious’.

## Data Availability

The data presented in this study are available on request from the corresponding author.

## Abbreviations

The following abbreviations are used in this manuscript:

ACMG	American College of Medical Genetics and Genomics
ATP	Adenosine Triphosphate
CSNB	Congenital Stationary Night Blindness
DNA	Deoxyribonucleic Acid
EDTA	Ethylenediaminetetraacetic Acid
ERG	Electroretinography
FAP	Familial Adenomatous Polyposis
*GPR179*	G Protein-Coupled Receptor 179
*GRK1*	G Protein-Coupled Receptor Kinase 1
HGMD	Human Gene Mutation Database
IRD	Inherited Retinal Disease
OCT	Optical Coherence Tomography
RP	Retinitis Pigmentosa
RPE	Retinal Pigment Epithelium
*SAG*	S-Antigen Visual Arrestin
*SLC24A1*	Solute Carrier Family 24 Member 1
*TRPM1*	Transient Receptor Potential Cation Channel Subfamily M Member 1
WES	Whole-Exome Sequencing
